# Arginine vasopressin hormone receptor antagonists in experimental autoimmune encephalomyelitis rodent models: A new approach for human multiple sclerosis treatment

**DOI:** 10.3389/fnins.2023.1138627

**Published:** 2023-03-14

**Authors:** Argelia Calvillo-Robledo, Cynthia Ramírez-Farías, Fernando Valdez-Urias, Erika P. Huerta-Carreón, Andrés Quintanar-Stephano

**Affiliations:** Departamento de Fisiología y Farmacología, Centro de Ciencias Básicas, Universidad Autónoma de Aguascalientes, Aguascalientes, Mexico

**Keywords:** multiple sclerosis, autoimmune disease, immune system, experimental autoimmune encephalomyelitis, arginine vasopressin hormone, arginine vasopressin receptors blockers

## Abstract

Multiple sclerosis (MS) is a chronic demyelinating and neurodegenerative disease that affects the central nervous system. MS is a heterogeneous disorder of multiple factors that are mainly associated with the immune system including the breakdown of the blood-brain and spinal cord barriers induced by T cells, B cells, antigen presenting cells, and immune components such as chemokines and pro-inflammatory cytokines. The incidence of MS has been increasing worldwide recently, and most therapies related to its treatment are associated with the development of several secondary effects, such as headaches, hepatotoxicity, leukopenia, and some types of cancer; therefore, the search for an effective treatment is ongoing. The use of animal models of MS continues to be an important option for extrapolating new treatments. Experimental autoimmune encephalomyelitis (EAE) replicates the several pathophysiological features of MS development and clinical signs, to obtain a potential treatment for MS in humans and improve the disease prognosis. Currently, the exploration of neuro-immune-endocrine interactions represents a highlight of interest in the treatment of immune disorders. The arginine vasopressin hormone (AVP) is involved in the increase in blood−brain barrier permeability, inducing the development and aggressiveness of the disease in the EAE model, whereas its deficiency improves the clinical signs of the disease. Therefore, this present review discussed on the use of conivaptan a blocker of AVP receptors type 1a and type 2 (V1a and V2 AVP) in the modulation of immune response without completely depleting its activity, minimizing the adverse effects associated with the conventional therapies becoming a potential therapeutic target in the treatment of patients with multiple sclerosis.

## 1. Introduction

### 1.1. Multiple sclerosis (MS)

Multiple sclerosis (MS) is an inflammatory disease of the central nervous system (CNS) characterized by the demyelination and destruction of the oligodendrocytes and axons of the neurons (Radandish et al., 2021) due to the infiltration of immune components through the blood-brain barrier (BBB) (Zlokovic, 2008; Bennett et al., 2010), leading to an a progressive loss of the myelin sheath. However, the onset and evolution of MS are clearly different between individuals (Reich et al., 2018; Wallin et al., 2019; Radandish et al., 2021). Some commonly shared symptoms can be categorized as motor, sensory, and cognitive dysfunctions. Recently, the contribution of temperature sensitivity to the aggressiveness of the symptoms has been recognized (Christogianni et al., 2018).

#### 1.1.1. Epidemiology

The epidemiology of MS remains a pivotal point, and its prevalence has been increasing in Europe, North America, and Australia, whereas in Sub-Saharan Africa, the reported cases are lower. In 2016, 2.2 million people were estimated to have MS worldwide based on the Global Burden of Diseases report (Wallin et al., 2019). Another study by Walton et al. (2020) estimated an increase in the number of cases to 2.8 million people (35.9 per 100,000 population); therefore, the prevalence of MS has increased worldwide.

Presently, the etiology of the disease is unknown; however, different factors, such as genetic, environmental, infections, smoking, obesity, immune system condition, and geographical latitude, are considered potential factors for developing MS. In addition, women are more at risk of developing MS (nearly three-quarters of patients with MS are women) (The International Multiple Sclerosis Genetics Consortium, Hafler et al., 2007; Ebers, 2008; Correa et al., 2016; Ruiz et al., 2019; Wallin et al., 2019; Voskuhl et al., 2020; Radandish et al., 2021; Kim and Patsopoulos, 2022). The first manifestation of the disease happens in patients in their 20 to 30 s and comprises the development of lesions in the brain’s white matter. Moreover, it can appear in the gray matter as an indicator of demyelination (early active white matter injuries), neuroinflammation, and glial activation (Reich et al., 2018; Walton et al., 2020; Radandish et al., 2021).

The clinical features of MS were categorized based on relapse frequency and pathological progression (Limmroth et al., 2011; Palle et al., 2017). The National Multiple Sclerosis Society is the Committee on Clinical Trials in Multiple Sclerosis that categorizes the clinical subtypes of MS (Lublin, 2014; Lublin et al., 2014). This categorization includes four principal subtypes: relapsing-remitting multiple sclerosis (RRMS), secondary progressive multiple sclerosis (SPMS), primary progressive multiple sclerosis (PPMS), and progressive relapsing multiple sclerosis (PRMS) (Lublin et al., 2014). Based on the occurrence rate, RRMS is the most prevalent subtype with approximately 80–85% of diagnosed cases and it is characterized by episodic exacerbations of symptoms, followed by transitory stability between “attacks” that steadily gets worse with the infiltration of immune components through the BBB into the CNS (Benveniste et al., 2014; Ruiz et al., 2019); the patients with RRMS are more susceptible to develop SPMS (Palle et al., 2017). SPMS is characterized by the progression of neurological disability, muscular weakness with troubled coordination, stiff or tight leg muscles, and occasional relapses and remissions (Thompson, 1997). A long relapse or remission is considered a predictor of disease progression in SPMS. On the other hand, PPMS is approximately 10–15% of progressive MS, with patients mostly men that are frequently older at onset compared to SPMS (Tremlett et al., 2008). Neuroaxonal deterioration and microglial activation are distinguishable in SPMS; nonetheless, there are some cases where the clinical features differ both immunologically and pathologically, and the diagnosed patients have poor prognosis owing to the high rate of mortality and advanced disability (Thompson, 1997; Frahm et al., 2021). In addition, PRMS is associated with progressive myelopathy and is considered a cryptical disease because it is frequently diagnosed as PPMS. However, until progressive neurological disability functions are detected, it is necessary to measure the period of relapse and perform neurological examination for accurate diagnosis (Tullman et al., 2004; Siger, 2022).

#### 1.1.2. Role of the immune system in MS development

The immune system (IS) plays an important role in the protection of our body and can recognize its own tissues from foreign molecules. The IS comprises lymphoid organs, cellular components, humoral factors, chemokines, and cytokines that respond to antigens that can harm the body (Shinde and Kurhekar, 2018; Verbsky and Routes, 2018). The IS is highly and tightly regulated; however, its disruption could induce diverse pathological disorders, such as allergies and asthma; subsequently, when the tolerance of the host is exceeded, the condition becomes an autoimmune disease (Anaya et al., 2016). Most autoimmune diseases such as rheumatoid arthritis, systemic lupus erythematosus, Hashimoto’s thyroiditis, Crohn’s disease, type 1 diabetes mellitus, and MS are considered chronic illnesses (Chamberlain et al., 2012; Anaya et al., 2016). Some of them can induce neuroinflammatory and neurodegenerative processes in the CNS, which occur when the integrity of the BBB is compromised (Zlokovic, 2008; Bennett et al., 2010). The BBB is considered a highly selective barrier between the cerebral capillary blood and interstitial fluid of the CNS (Kadry et al., 2020; Schreiner et al., 2022), and helps keep harmful substances from reaching the brain as pathogens, toxins, and some drugs, as well as prevents the entry of IS components (Daneman, 2012; Kadry et al., 2020; Knudsen et al., 2022). The principal constituent of the BBB is the endothelial cells, which provide protection and structural stability to the blood vessels on tight junctions (the liner sheets pericytes, and astrocytes that ensheath the blood vessels and restrict the substances entering the brain) (Daneman, 2012). The breakdown of the BBB promotes its permeability, permitting the entrance of immune molecules and lymphocytes, inducing autoreactive conditions in the blood-spinal cord barrier and BBB; as a consequence, damage and destruction of the myelin sheath increases, inducing neurodegenerative disorders such as Alzheimer’s disease, Parkinson’s disease, and MS (Engelhardt, 2006; Daneman, 2012; Heneka et al., 2015; Wang et al., 2015; Knudsen et al., 2022). Similarly, neuroinflammation promotes the entry of IS components through the BBB and blood-spinal cord barrier (Arima et al., 2013; Milovanovic et al., 2020) into the CNS; therefore, an increase in the barriers’ permeability induces the interaction of pro-inflammatory cytokines such as the interleukins 1β and 17A (IL-1β and IL-17A), and tumor necrosis factor α (TNFα), which activates the downregulation of tight junctions in the endothelial cell barriers (Arima et al., 2013). IL-17A has been associated with the loosening of both barriers, as shown by *in vivo* and *in vitro* assays, and it is related to the production of reactive oxygen species by nicotinamide adenine dinucleotide phosphate (NADPH) and xanthine oxidase action, which are related to increase in the endothelial cells’ permeability, causing a decrease in the occluding protein, zonula occludens-1 in *in vitro* assays (Arima et al., 2013). In contrast, hyperactivity of sensitized lymphocytes induces the proliferation and secretion of IL-17 in MS (Ruiz et al., 2019; Volpi et al., 2019). In addition, under normal conditions, T regulatory (Treg) cells alter and breakdown the balance in IS responses, which are accompanied MS (Pot et al., 2011). Immune cells such as T and B lymphocytes, as well as macrophages and innate immune cells, promote the pathophysiology of the disease (Radandish et al., 2021). Myelin antibodies from B cells induce the loss of the myelin sheath. Furthermore, studies have demonstrated the presence of immunogobulins IgG and IgM in patients with acute and chronic MS (Duffy et al., 2014).

**FIGURE 1:**
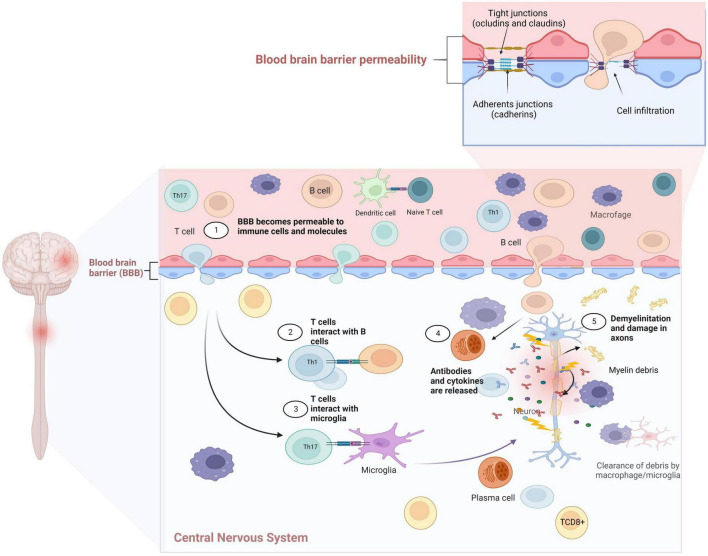
Dendritic cells can activate autoreactive T cells and promote clonal expansion in the lymph nodes. T cells differentiate to Th1 and Th17, which releases proinflammatory cytokines (IFN-γ, IL-17). These cells invade the central nervous system (CNS) and are reactivated, inducing a positive loop that continues to promote the autoactivation of autoantibodies and autoreactive T cells. The presence of these autoreactive Th1 and Th17 cells, cytotoxic T lymphocytes and B lymphocytes in the CNS, with astrocytes and activated microglia will lead to increased production of inflammatory cytokines, nitric oxide (NO), glutamate-mediated excitotoxicity, autoantibody production, and cytotoxicity, leading to damage and destruction of the myelin sheaths and axons. Created with BioRender.com.

Similarly, the innate IS is related to the recognition, processing, and destruction of pathogens in the body (Nicholson, 2016); however, it has also been associated with the development and progression of MS, antigen-presenting cells (APCs), and proinflammatory cytokines, such as IL-1α, IL-1β, and IL-6, considered as the main mediators of signal pathways related to the BBB disruption (Jara et al., 2006; Chastain et al., 2011). The entry of components of the IS through the BBB is shown in Figure 1. The major treatments for MS focus on CD4^+^ T cells regulation; however, the impact of CD8^+^ T cells and B-cells reveals that they also play an important role in the disease inflammatory process and progression (Lassmann and van Horssen, 2011), suggesting that the pathogenic response of each patient is critical for determining an optimal treatment approach (Lehmann-Horn et al., 2013).

##### 1.1.2.1. Role of CD4^+^ T cells in MS

Lymphocytes are classified according to their differentiation and recognition of peptides presented by the major histocompatibility complex (MHC). CD4^+^ T lymphocytes are capable of recognizing MHC class II molecules, and they participate in the adaptive IS response by presenting on their surface specific antigen recognition receptors (TCR), which is critical for their efficient activation. Once they migrate to the thymus, they acquire their specific TCR and can recognize self and strange antigens; however, when their tolerance limit is exceeded or compromised, the probability of developing autoimmune diseases increases (Chitnis, 2007). In MS, the participation of autoreactive CD4^+^ T cells is considered one of the most important immunological components in the disease development. These cells are associated with the breakdown and increase in the permeability of the BBB through the secretion of Th1 cells of interferon gamma (INF-γ), IL-2, TNF-α, and lymphotoxin. These CD4^+^ T cells express α4 integrin (VLA-4) on their surface, which is required for their entry across the BBB (Baron et al., 1993; Schreiner et al., 2022). Activated reactive CD4^+^ T cells can be found in the cerebrospinal fluid (CSF) of patients with MS in contrast to healthy individuals. This evidence suggests that most therapies focus on the regulation of CD4^+^ T cells (Chitnis, 2007). Peeters et al. (2017) reported that cytotoxic CD4^+^ T cells, known as CD4^+^ CTL (CD4^+^ CD28^null^) increase the inflammatory conditions mediated by INF-γ, and this cytotoxicity increases the severity of MS (Peeters et al., 2017). Other studies have revealed that subsets of CD4^+^ T cells are related to several regulatory factors, including the microenvironment, transcription factors, and stimulatory or inhibitory cytokines, as it is in the case of Th1 and Th17 lymphocytes, which are considered inducers of MS in the immunopathogenesis. Nonetheless, transforming growth factor β1 (TGF-β1) participates in the suppression of transcription factors such as T-bet and GATA3, which are closely associated with Th1 and Th2 cell differentiation (Jadidi-Niaragh and Mirshafiey, 2011). In experimental autoimmune encephalomyelitis (EAE), TGF-β cells induce Th2 differentiation, as well as the secretion of IL-2, IL-10, and TGF-β, and regulate the Th1 phenotype related with MS disease (Wolf et al., 1996).

##### 1.1.2.2. Role of CD8^+^ T cells in MS

CD8^+^ T cells are associated with MHC class I (endogenous peptides expressed in nucleated cells) and are essential for maintaining immunological homeostasis by detecting and eliminating infected and abnormal cells. Under physiological conditions, CD8^+^ T cells are capable of differentiating self from strange antigens, and mediate cytotoxic effects by secreting cytokines and enzymes (Friese and Fugger, 2005; Zhang and Bevan, 2011). In MS, CD8^+^ T cells become autoreactive and can induce the infiltration of the immune components into the BBB, which is considered a critical factor in the disease pathogenesis (Galea et al., 2007). B and T lymphocytes, as well as plasma cells and antibodies, have been related to the infiltration and promotion of brain and spinal cord damage, mainly by the immunological components that are involved (Friese and Fugger, 2005). APCs such as dendritic cells (DCs), are involved in the activation, invasion, and clonal expansion of CD8^+^ T cells into the CNS by the stimulus produced by viruses or bacteria during an infection; therefore, the processed molecules are presented (MHC Class II) to CD4^+^ T cells, permitting the DCs to activate CD8^+^ T cells. CD8^+^ T cells, once activated, differentiate into autoreactive cytotoxic CD8^+^ T cells. In this stage, they have the ability to enter the CNS across the BBB using P-selectin glycoprotein ligand-1, α4β1 integrin (Very late antigen-4, VLA-4), and lymphocyte function-associated antigen 1. In addition, cytotoxic CD8^+^ T cells can interact with microglial cells and macrophages by recognizing co-stimulatory CD80/86, CD40, and MHC class I, and inducing cytotoxic CD8^+^ T cells to multiply and expand clonally and recognize their target antigens through MHC class I in the oligodendrocytes and neurons (Ramakrishna et al., 2004; Friese and Fugger, 2005). In MS acute and chronic lesions, cytotoxicity of CD8^+^ T cells is mediated by the induction and release of pro-inflammatory cytokines, such as INF-γ, TNF-α, lymphotoxin, granzyme, and perforin (Patel and Balabanov, 2012). These molecules are toxic to the oligodendrocytes and damage the axonal myelin sheath. These findings have been observed in animal models and in patients diagnosed with MS (Huseby et al., 2012).

On the other hand, in human MS, some treatments are associated with the depletion of the membrane antigen in CD4^+^ and CD8^+^ T cells by Campath-1H (anti-CD25 monoclonal antibody), which induces a decrease in the number of relapses and lesions, showing improvement in neurological responses (Friese and Fugger, 2005).

##### 1.1.2.3. Role of pro-inflammatory cytokines in MS

In the IS, cytokines are responsible for cell differentiation, inflammation, cell activation, and immunoglobulin production, as well as in the development of several pathological disorders such as cancer and autoimmune diseases (Chitnis, 2007). In MS, the major mediators of the disease progression are the Th1 cells, which produce TNF-α, IL-2, and INF-γ, whereas Th2 cells secrete the interleukins IL-4, IL-5, IL-10, and IL-13, which are considered MS protective factors because of their participation in the regulation and suppression of Th17 cells (Park et al., 2005). In addition, Th3 cells that produce TGF-β are associated with the differentiation and regulation of Treg cells (CD4^+^CD25^+^Foxp3^+^). Th17 cells, which are highly present in the CSF and brain lesions, are responsible for inducing the secretion of pro-inflammatory cytokines such as IL-17, INF-γ, IL-16, IL-21, IL-22, IL-23, and TNF-α, which are closely associated with the progression of MS owing to their pro-inflammatory features (Jadidi-Niaragh and Mirshafiey, 2011; Pot et al., 2011; Ruiz et al., 2019). The categorization of inflammatory cytokines in the progression of MS has been documented. They participate in the regulation, modification, and release of cytokines and chemokines, enabling the activation or differentiation of multiple cells such as lymphocytes, plasma cells, and APCs (Jadidi-Niaragh and Mirshafiey, 2011). In the case of Th17 cells, an increase in inflammation and neutrophil secretion in the CNS, as well as the activation of metalloproteinase-3, can induce the BBB breakdown and increase macrophage migration into the CNS, causing damage to the foci and injury to the myelin sheath and axons (Jadidi-Niaragh and Mirshafiey, 2011).

In addition, once the immune cells receive specific signals, the production and secretion of cytokines is dependent on the cell type that can produce them (Chitnis, 2007); thus, IL-2, TNF-α, and INF-γ are produced by Th1 cells, whereas the secretion of TGF-β, IL-6, and IL-23 induces the differentiation of Th17 cells (Lovett-Racke et al., 2011; Lowther and Hafler, 2012; Lee, 2018).

##### 1.1.2.4. Role of Foxp3 and regulatory T (Treg) cells in MS

Treg cells are one of the most important regulators of the IS. They participate in the control of self-reactive antigens. Therefore, alterations in the regulation and tolerance of these cells promote a disturbance and modify their regulatory functions (Kondělková et al., 2010). In the CNS, Treg cells participate in the maintenance of neuroprotection and cognitive functions such as memory and learning (Lowther and Hafler, 2012). Their regulatory potential is associated with their specific phenotypic expression of CD4^+^/CD25*^high^*/CD127*^low–neg^*, and in severe cases, the presence of the natural transcription factor, Foxp3, is required for their development and adequate function. To achieve this, Treg cells secrete specific cytokines that are involved in several functions, such as the regulation and differentiation of CD4^+^, CD8^+^, B cells, natural killer (NK) cells, and APCs (Verma et al., 2021). In autoimmune diseases, depletion of CD25^+^ can induce abnormalities and affect several organs in a Balb/c mice model, demonstrating the immunomodulatory role of these cells in the development of autoimmune disorders (Dominguez-Villar and Hafler, 2018; Verma et al., 2021). Several studies have demonstrated that mutations in Foxp3 in both humans and mice are closely associated with high aggressiveness and immune dysregulation of diseases related to polyendocrinopathy, enteropathy, and X-linked syndrome (Baud et al., 2001; Wildin, 2002).

Moreover, the loss of the Treg equilibrium in MS has been associated with an increase in CD4^+^ differentiation to Th17 cells, which are induced by IL-6 and TGF-β. This cellular phenotype is considered principal encephalitogenic cells (Th17) in EAE. Similarly, TGF-β induces the differentiation of pTregs; therefore, when the balance between Th17 and Treg cells is broken, autoimmune diseases develop (Lovett-Racke et al., 2011; Lowther and Hafler, 2012; Lee, 2018). In addition, Jamshidian et al. (2013) demonstrated an inverse correlation between Th17 and Treg cells in healthy controls and patients with MS during relapses, demonstrating that the disruption of phenotypic cells influenced the prognosis of the disease (Jamshidian et al., 2013).

In addition, Treg cells and their role in MS have been related to the participation of DCs, which promote the activation of Tregs and functional encephalitogenic cells in the CNS (Stasiolek et al., 2006). Similarly, the regulation of the inflammation and autoimmunity of the Type 1 T regulatory cells (Tr1) with Tregs, suppresses hyperactivation and autoimmune response; however, Tr1 cells co-express CD49b, LAG-3, and CD226 on their surface and do not express the Foxp3 factor. Tr1 cells promote immunosuppression in EAE models through the release of IL-10 and TGF-β and have been linked to the differentiation of tolerogenic DCs, inducing an increase in the anti-inflammatory processes mediated by IL-10 (Pot et al., 2011; Ruiz et al., 2019).

##### 1.1.2.5. Role of regulatory B cells in MS

B cells participate in adaptive immunity and are responsible for humoral immune response. Under healthy conditions, B cells are found in relatively low levels within the brain (CD20^+^ CD23^+^ cell phenotype) (Chitnis, 2007; van Langelaar et al., 2020). In the CNS, chemokines and cytokines are responsible for regulating the mobilization of lymphocytes and APCs. For instance, the presence of CXCL13 increases the recruitment of B cells into the CSF in autoimmune diseases such as MS and chronic rheumatoid arthritis (RA) (Kowarik et al., 2012; Armas-González et al., 2018). In MS, B cells are associated with the secretion of autoreactive antibodies when they differentiate into plasma cells that participate in sheath demyelination and injury, and regulate the action of the complementary system and act as a potent promoter of pro- and anti-inflammatory cytokines in the CNS (Lehmann-Horn et al., 2013). The presence of oligoclonal antibodies in the CSF can be related to the aggressiveness and poor prognosis of the disease. In contrast, in the EAE model, the release of autoantibodies by plasma and B cells that are expressed on the surface of CD20 during the stages of maturation, has been found in cortical lesions in the CNS (Weber et al., 2010). The use of anti-CD20 antibodies can inhibit the maturation of B cells and promote their apoptosis, suggesting that anti-CD20 antibodies may be effective as a treatment for MS (Lehmann-Horn et al., 2013). In addition, in the myelin oligodendrocyte glycoprotein (MOG)-induced EAE model, the use of anti-CD20 antibodies decreased the levels of Th1 and Th17 cells in the CNS (Weber and Hemmer, 2009). The deficiency of B cells caused by the deletion of their μ chain transmembrane region showed that mice recovered from the EAE acute phase, suggesting that the deletion of the μ chain of the B cells is involved in the severity of EAE. In addition, B cells can play a role in EAE and MS through the regulation of differentiation of both Th1 and Th2 cells (Wolf et al., 1996).

Furthermore, B cells act as APCs for the activation of T cells, and T cells can secrete pro-inflammatory cytokines such as INF-γ, IL-6, and TNF-α associated with the transport of B cells into the CNS and have been linked to the activation of plasma cells in MS, closely related to the production, secretion, and infiltration of autoantibodies into the BBB (Kowarik et al., 2012).

##### 1.1.2.6. Role of NK cells in MS

The principal function of NK cells is to detect and eliminate viruses and tumor cells. These cells represent 10–20% of the peripheral blood mononuclear cells (PBMCs) and constitute an essential protection line of the lymph nodes and CSF (Zamai et al., 2007). Several studies have demonstrated the role of NK cells in the modulation of the IS and MS in the early stages mainly in RRMS. NK cells secrete IL-17A and IL-17F that promote the permeability of the BBB in MS (Jadidi-Niaragh and Mirshafiey, 2011) and induce the cytotoxicity of autologous oligodendrocytes, inducing the inflammatory process; however, in the acute phase, NK cells can reduce damages and inflammations through the control of autoreactive T cells and microglia. On the other hand, NK cell deficiency has been associated with an increase in autoimmune episodes in MS during disease progression (Chanvillard et al., 2013; Kucuksezer et al., 2021). In addition, studies by Gross et al. (2016) demonstrated the role of NK cells in T-cell activity through the control and modulation of Treg cells and DCs in patients with MS (Gross et al., 2016). Moreover, the depletion of NK cells is related to a higher severity and exacerbation of symptoms in C57BL/6 mice immunized for EAE (Chanvillard et al., 2013). Some studies have attributed the dual functions of NK cells to their surface markers (CD56^+^ and CD16^+^). The pathogenesis of MS increases with CD56^bright^ NK cells, as these cells promote intensive secretion of cytokines (INF-γ, TNF-α, and IL-10) and adaptive immune response, whereas CD56^dim^ is closely associated with cytolysis. These immunoregulatory functions have been reported in clinical trials in patients (Kucuksezer et al., 2021; Ahmadi et al., 2022). Some drugs, such as daclizumab, participate in the control of MS because of their association with the inhibition of the marker CD56^brigh^ (Ruiz et al., 2019).

##### 1.1.2.7. Role of DCs in MS

DCs are the most efficient APCs owing to their ability to take up several types of antigens and pathogens to generate MHC-peptide complexes and then migrate from the sites of antigen acquisition to secondary lymphoid organs, which can physically interact with and stimulate T lymphocytes. DCs contribute to the initiation of immune responses and differentiation of naïve T cells by specific stimulation, which is more efficient than the ability of B cells and macrophages as APCs (Inaba and Steinman, 1984). Changes in the BBB secondary to peripheral activation of DC-mediated myelin-reactive T cells promote CNS perivascular infiltration, with the production and secretion of pro-inflammatory cytokines (Nuyts et al., 2013). Furthermore, plasmacytoid DCs have been found in the CSF, leptomeninges, and white matter lesions, as well as in the relapse phase, with an increase in plasmacytoid DCs compared to remission (Stasiolek et al., 2006; Nuyts et al., 2013).

#### 1.1.3. Types of treatments for MS

Currently, there is no cure for MS; however, new therapeutic advances for the control of the disease have been achieved. These therapies, called disease-modifying therapies, improve the disease prognosis and patients’ quality of life. They include drugs, stem cells, and monoclonal antibodies (see in MS Society home page). A deep knowledge of the mechanisms of action, pharmacokinetics, efficacy, security profiles, and the possible adverse effects is important for consideration because of the great variability in the response of each patient; thus, it is necessary to adapt the therapy to the different conditions of the patients (Callegari et al., 2021). The following tables describe the different types of disease-modifying therapies (DMTs) approved for MS (Tables 1–6).

**TABLE 1:** Disease-modifying therapies (DMTs) approved for relapsing-remitting multiple sclerosis (RRMS).

DMT compound	DMT type	Disease treatment	Mecanism of action	Regimen posology	Administration route	Secondary effects	References
Interferon β	Cytokine	1st line for RRMS and SPMS	Mediates an anti-inflammatory and regulatory response by downregulating the immune recognition molecules, inhibition Th1–Th2 shift.	Variable	Subcutaneous	Injection-site reactions, flu symptoms, anemia, leukopenia, thrombocytopenia, depression, hepatotoxicity, skin necrosis, thrombotic microangiopathy (TMA).	Goldenberg, 2012; Martin et al., 2016; Hauser et al., 2020; Bayer Inc., 2021; Callegari et al., 2021
Glatiramer acetate	Synthetic amino acid copolymer	1st line for RRMS	Binds to MHC and competing with myelin for antigens that allow its antigenic presentation to T cells, in addition to an induction of suppressor Th2 cells that migrate to the brain and express IL-10 and TGF-β.	20 mg/day or 40 mg 3 weeks	Subcutaneous	Injection-site reactions, skin necrosis, flu-symptoms, lipoatrophy, vasodilation, rash, anemia, leukopenia, thrombocytopenia, depression, hepatotoxicity.	Arnon and Aharoni, 2004; Racke and Lovett-Racke, 2011; Hauser et al., 2020; Callegari et al., 2021

**TABLE 2:** Monoclonal antibodies.

DMT compound	DMT type	Disease treatment	Mecanism of action	Regimen posology	Administration route	Secondary effects	References
Alemtuzumab	Monoclonal antibody	1st line for RRMS	T cells depletion by interaction with CD52 (molecule expressed in T cells, B cells, natural killer cells and monocytes)	12 mg/day for 5 days after 1 year, 12 mg/day for 3 days.	Intravenous	Autoimmune hemolytic anemia, alveolar hemorrhage, nephropathy, and cerebrovascular accident (CVA) simultaneously.	Cox et al., 2005; Alnahdi et al., 2020; Hauser and Cree, 2020; Callegari et al., 2021; Sanofi-Genzyme, 2021; Knudsen et al., 2022
Natalizumab	Monoclonal antibody	2nd line for RRMS	Binds to α4/β1 integrin, expressed by lymphocytes and blockade its interaction with vascular cell adhesion molecule-1 (VCAM-1). Prevents leukocyte transmigration across the endothelium into inflamed parenchymal tissue.	300 mg every 4 weeks.	Intravenous	Progressive multifocal leukoencephalopathy (PML), infusion reactions, urticaria, headache, mild leukocytosis, and anaphylaxis.	Hauser and Cree, 2020; Callegari et al., 2021; Kougkas et al., 2022; Morrow et al., 2022
O Ofatumumab	Monoclonal antibody type 1	1st line for RRMS and SPMS	Depletes CD20^+^ cells, binding to the small loop epitope near the surface induces high complement-dependent cytotoxicity.	20 mg every 4 weeks.	Intravenous	Nasopharyngitis, headache, infusion site reaction, urinary tract infection and/or upper respiratory infection.	Lin, 2010; Klein et al., 2013; Engelberts et al., 2016; Semple et al., 2019; Hauser and Cree, 2020; Hauser et al., 2020; Callegari et al., 2021
O Ocrelizumab	Monoclonal antibody type 1	1st line for RRMS and PPMS	Selectively targeting CD20-expressing B cells.	600 mg every 6 months.	Intravenous	Infusion reactions, infections (respiratory tract, hepatitis B, PML), hypogammaglobulinemia, neutropenia.	Klein et al., 2013; Hauser et al., 2020; Callegari et al., 2021; Hoffmann-La Roche Limited, 2022

**TABLE 3:** Oral immunomodulatory therapies.

DMT compound	DMT type	Disease treatment	Mecanism of action	Regimen posology	Administration route	Secondary effects	References
Dimethyl fumarate (dmf)	Fumaric acid esters	1st line for RRMS	Inhibition of NFκB of activated B cells pathway, downregulation of aerobic glycolysis a pyroptosis in activated myeloid and lymphoid cells. Reduce CD8^+^ T cells and CD4^+^. Shift Th cells to a pro-inflammatory state. Apoptosis of T cells and dendritic cells.	240 mg twice daily	Oral	Gastrointestinal tract reactions (diarrhea, nausea, and abdominal pain), lymphopenia, pruritus, PML, hepatotoxicity, anaphylaxis	Ghoreschi et al., 2011; Gold et al., 2012; Mills et al., 2018; Hauser et al., 2020; Callegari et al., 2021; Majkutewicz, 2022
Monomethyl fumarate (mmf)	Fumaric acid esters	RRMS	Protects astrocytes by activate Nrf2. Decrease monocytes migration across BBB by reduce the expression of vascular cell adhesion. Reduce CD8^+^ and CD4^+^ T cells. Induce dendritic cells apoptosis and differentiation, reducing IL-12.	190 mg twice daily	Oral	Abdominal pain, flushing, nausea, diarrhea, rash, redness, itching, PML, swelling (face, lips, tongue, or mouth), liver failure	Moharregh-Khiabani et al., 2009; Mazzola et al., 2019; Hauser et al., 2020; Berger et al., 2021
Diroximel fumarate	Fumaric acid esters	1st line for RRMS and PPMS	Inhibit KEAP1-Nrf2 interaction.	462 mg twice daily	Oral	GI system disorders included nausea, diarrhea, upper abdominal pain, vomiting, constipation, flatulence, gastroesophageal reflux disease.	Palte et al., 2019; Hauser and Cree, 2020
Teriflunomide	Oral therapy	1st line for RRMS	Reduction of the activated b and T cells by inhibiting pyrimidine biosynthesis.	14 mg/day	Oral	Gastrointestinal tract reactions (diarrhea and nausea), headache, paresthesia, Stevens-Johnson syndrome, infections, peripheral neuropathy, hypertension	O’Connor et al., 2011; Bar-Or et al., 2014; Hauser and Cree, 2020; Callegari et al., 2021

**TABLE 4:** Sphingosine 1-phosphate receptor modulators (S1PR).

DMT compound	DMT type	Disease treatment	Mecanism of action	Regimen posology	Administration route	Secondary effects	References
Fingolimod	S1PR modulators	2nd line for RRMS	Binds to S1PRs and induce their internalization and degradation, preventing the egress of lymphocytes from lymph nodes.	0.5 mg/day	Oral	Gastrointestinal tract reactions, infections, headache, hepatotoxicity, backpain, cough, PML, macular edema	Yanagawa et al., 1998; Kappos et al., 2010; Hauser and Cree, 2020; Callegari et al., 2021
Siponimod	S1PR modulators	1st line for SPMS and RRMS	Binds to S1PR1 and reduces phosphatidylcholine, TNF-α, IL-6. *Vía* S1PR5 maintains BBB integrity, preserves OLGs and myelinated axons.	2 mg/day	Oral	Hypertension, headache, macular edema, hepatotoxicity, infections, posterior reversible encephalopathy syndrome (PRES).	Gentile et al., 2016; Cohan et al., 2020; Hauser and Cree, 2020; Callegari et al., 2021
Ozanimod	S1PR modulators	1st line for RRMS	Reduces T and B cells circulating by modulates S1PR1	0.92 mg/day	Oral	PRES, hypertension, hepatotoxicity, infections, orthostatic hypotension, back pain, leukopenia, macular edema.	Scott et al., 2016; Cohen et al., 2019; Callegari et al., 2021

**TABLE 5:** Cladribine.

DMT compound	DMT type	Disease treatment	Mecanism of action	Regimen posology	Administration route	Secondary effects	References
Cladribine	Induction therapy	2nd or 3rd line for PPMS and RRMS	Induce cellular metabolism disruption	1.75 mg/kg for 4–5 days cycle with a total of 2 cycle 1 Year apart from each other.	Oral	Headache, infections, cancer, leukopenia	Carson et al., 1983; Giovannoni et al., 2010; Hauser and Cree, 2020; Callegari et al., 2021

**TABLE 6:** Third line therapies.

DMT compound	DMT type	Disease Treatment	Mecanism Of Action	Regimen Posology	Administration Route	Secondary Effects	References
Mitoxantrone (Mx)	Anthracenedione derivate/DNA intercalator	2nd or 3rd line for SPMS and RRMS	Reduce B cells population (CD4^+^), inhibits T helper cell function (as antigen presentation, antibody-dependent demyelination and complement mediated myelinolysis), inhibits demyelinating activity of macrophages and enhance T cell suppressor activity.	12 mg/m^2^ every 3 months, max cumulative dose 120–140 mg/m^2^	Intravenous	Nausea, vomit, urinary tract and upper respiratory tract infections, alopecia, leukopenia and rarely cardiotoxicity, myelosuppression, and leukemia.	Gonsette, 2003; Goldenberg, 2012; Martinelli Boneschi et al., 2013; Hauser and Cree, 2020; Callegari et al., 2021
Autologous hematopoietic stem cell transplantation (ahsct)	Immune reconstitution	–	Bone marrow repopulation and immune reconstitution after ablation (chemotherapy high dose).	NA	Intravenous	Viral reactivation, neutropenic fever	Hauser and Cree, 2020; Callegari et al., 2021

### 1.2. Experimental autoimmune encephalomyelitis (EAE) models

The basic mechanisms underlying the pathophysiology of MS have been recognized in EAE animal models, which are considered good models for studying several signs and trial treatments to alleviate or control MS (Constantinescu et al., 2011). This model was first reported in 1933 by Rivers et al. (1933) in which acute disseminated encephalomyelitis was induced in monkeys. This demonstrated the potential of the model to recreate the immunological and pathophysiological processes of MS. Even though MS presents high heterogeneity among the affected population, the similarities of EAE in murine models has made it possible to observe sequentially the various stages of the disease during neuroinflammation; both of them are characterized by immunological cell infiltration in the CNS causing the subsequent demyelination of oligodendrocytes, axonal dysfunction, and disability depending on the selected model. However, the main difference between MS and EAE is the external immunization of the animals whereas in humans there are multiple autoantigens but they are not induced, as well as the timeline of the clinical and pathophysiological course in both MS and EAE. Therefore, these differences and similarities show the potential use of *in vivo* models in preclinical trials, with the main objective of finding the best model that can replicate the pathophysiological conditions of the disease, consequently their study is key to understanding the disease, as well as the creation of possible therapeutic targets for patients with MS (Mix et al., 2010; Constantinescu et al., 2011; Lemus et al., 2018).

Immunization with myelin antigens isolated from the brains and spinal cord oligodendrocytes of several species of animals emulsified with complete Freund’s adjuvant (CFA) has been used to induce EAE including the antigens proteolipid peptide (PLP), myelin basic protein (MBP), myelin oligodendrocyte, MOG, and specific recombinant fragments of myelin (Bettini et al., 2009; Constantinescu et al., 2011; Greer et al., 2020; Petersen et al., 2021). On the other hand, the more acceptable models by their reproducibility are the small encephalitogenic peptides, such as MBP, MOG, and PLP. Differences in the onset and development of EAE are closely associated with the animal species and microenvironment where the immunological factors are localized (Gerdoni et al., 2007). The methodology and variants of every EAE subtype are focused on the autoantigen target and genetic susceptibility of the experimental models (Kawakami et al., 2004; Viñuela-Berni et al., 2020). For instance, the Lewis strain is considered an animal model that is very useful because of its susceptibility to developing inflammatory diseases (RA and EAE), whereas Fischer (F344/N) is resistant to developing it and it is more useful in demonstrating non-inflammatory diseases or resistance to inflammatory diseases (Patchev et al., 1993; Wilder et al., 2000). The pathophysiological components of MS are similar to those of EAE reported in some studies, such as genetic susceptibility to MHC II, Kim and Patsopoulos (2022), lesions in the white matter related to autoreactive cells (Costantino et al., 2008), and gray matter associated with axonal degeneration. Degeneration, inflammation, and demyelination of oligodendrocytes are characteristics of EAE and MS (Patel and Balabanov, 2012; Lemus et al., 2018). Leukocyte transport through the BBB in RRMS were found in EAE (Ruiz et al., 2019). The most important goal is achieving successful and appropriate treatments for the patients because the physiopathology changes between one patient and another are heterogeneous, which hinders therapeutic success.

The classical EAE is based on the inductive effects of several types of animal models, such as C3H.SW (H-2*^b^*) immunized with recombinant rat MOG (rMOG), and characterized by inflammation that is restricted to the meninges T cells, showing a wide range of Th17/Th1 ratios that induces differential regulation of CNS and spinal cord autoimmunity (Stromnes et al., 2008), whereas in C57BL/6 mice (immunized rMOG) laquinimod modulates myelin antigen-specific B cell immune responses and diminishes both the development of meningeal B cell aggregates and disability progression in EAE, demonstrating their potential successful for preclinical treatment of MS (Stromnes et al., 2008; Varrin-Doyer et al., 2016). In another model, the C3HeB/Fej (H-2k) strain mice showed ataxia, spasticity, and hyper-reflexivity associated with the infiltration of CD4^+^ T cells and autoreactive microglia (Stromnes et al., 2008). In addition, transgenic mice (DRB1*1501) developed spontaneous EAE associated with PLP, which is considered the most abundant protein in the CNS myelin, causing the overexpression of *PLP1* induced myelin defects and promotion of demyelination, axonal damage, and accumulation of PLP in the oligodendrocytes (Mastronardi et al., 1993; Knudsen et al., 2022; Ruskamo et al., 2022). On the other hand, the brain and spinal cord of guinea pigs have been used as encephalitogens to replicate acute self-limiting or chronic relapsing-remitting diseases (Constantinescu et al., 2011). Some studies used the SJL/J mice to replicate the MS disease. In 1991, Whitham et al. (1991) developed a relapsing EAE model using MBP and PLP (PLP139-151) in CFA to induce acute EAE at 9–12 days post-inoculation, suggesting that the background of a biological model may be a predominant factor in myelin Ag response in patients with MS. The classification of clinical signs in EAE models is described by the progressive weakness in the extremities, beginning with a loss of tail tone, followed by the limp tail and hind leg weakness, and subsequently resulting in a limp tail and complete paralysis of the hind legs. The animal suffers motor paralysis, bladder overflow, and constipation in advanced disease stage in which euthanasia is recommended to protect the animals from suffering (Huseby et al., 2012).

Furthermore, both acute and chronic EAE are focused on the replication of clinical and histopathological signs of human MS. The acute EAE is the most used model because of the short duration of the acute phase of the clinical and pathophysiological symptoms (15 days post-immunization), and the short-term evaluation of the experimental results. One study by Elo et al. (2021) reported that the use of Lewis males in the chronic EAE model induced by CFA supplemented with *Mycobacterium tuberculosis* causes a short acute phase of inflammatory signs and BBB breakdown (day 14), followed by chronic clinical symptoms of EAE, inducing the chronic features of the disease 3 months post-immunization; thus, replicating the signs of chronic human MS (Elo et al., 2021, 2019).

#### 1.2.1. Treatments of EAE

Currently, the optimal targets of specific EAE therapies are of great experimental interest, many of which have been extrapolated for human benefit. The targets are based on neuroimmune factors that can modulate the production and differentiation of immunological components (Peine et al., 2014). The trial treatments include different approaches, such as the use of the hormone estradiol (E2) because of its immunomodulatory and neuroprotective properties (Gold and Voskuhl, 2009; Ghareghani et al., 2021) and its association with the development of several autoimmune diseases. MS has been related to the regulation of TNF-α (Peón and Terrazas, 2018), which promotes BBB permeability (Schreiner et al., 2022). However, several studies have demonstrated that the use of estriol improves disease symptoms in patients with MS, and that active and adoptive EAE were controlled by the administration of estriol and 17β-estradiol treatment in SJL, C57BL/6, B10.PL, and B10.RIII mouse strains (Subramanian et al., 2003). In addition, it was shown that oral administration of ethinyl estradiol inhibited the recruitment of inflammatory cells into the CNS in SJL mice (Subramanian et al., 2003; Gold and Voskuhl, 2009).

**FIGURE 2:**
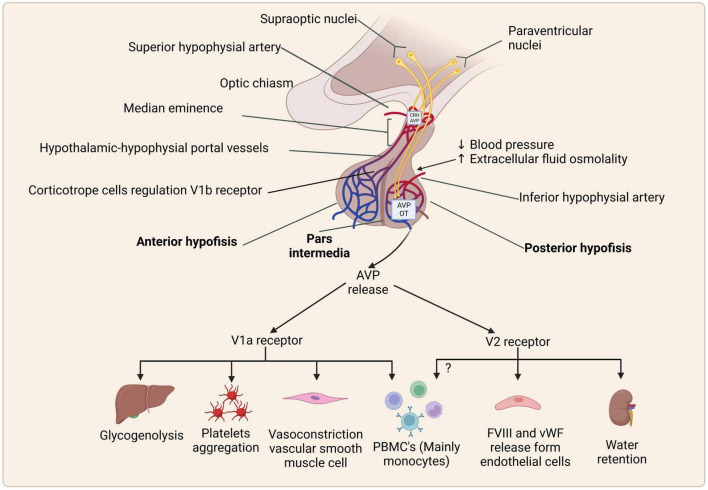
Schematic representation of synthesis, storage, and effects of arginine vasopressin (AVP) *via* AVP receptors and their expression. Created with BioRender.com.

The above information on the role of estrogens in the regulation of EAE clinical symptoms suggests that the endocrine system may be a potential option for treating EAE rats and patients with MS. An integral approach to study the role of the endocrine system on the regulation of the IS is through the neuroendocrine-immune (NEI) system, a regulatory network formed by the nervous, endocrine, and immune systems, which carries out a reciprocal regulation to maintain homeostasis in the host through the involvement of signaling molecules, such as neurotransmitters, hormones, and cytokines. The accumulating evidence in the last decade clearly demonstrates the importance of NEI network in the regulation of physiological homeostatic mechanisms, particularly immunomodulation functions (Gaillard, 1995; Haddad et al., 2002). Thus, EAE could be a good model for studying scarcely known neuro-immune-endocrine interactions due to hormonal factors that could influence the onset and development of EAE (Peón and Terrazas, 2018; Ghareghani et al., 2021). On the other hand, the hypothalamic hormone, arginine vasopressin (AVP), has been shown to play an important role in IS control. Experiments on Wistar and Lewis rat strains, subjected to neurointermediate pituitary lobectomy (NIL) induced a permanent decrease in AVP and oxytocin (OXT) serum levels. Immune studies in these animals showed that innate, humoral, and cell mediated immune responses were decreased (not suppressed) (Quintanar-Stephano et al., 2012; Quintanar-Stephano et al., 2019). Experiments in NIL Lewis rats immunized for EAE, showed that NIL animals developed just a mild clinical symptom of EAE, whereas desmopressin administration (an agonist of V2 AVP receptors) restored the susceptibility of these animals to EAE (Quintanar-Stephano et al., 2016, 2012, 2018). Similarly, AVP is involved in some CNS disorders, such as stroke, brain edema, ischemia, and modulation of the IS in EAE (Hedna et al., 2014; Zeynalov et al., 2015; Viñuela-Berni et al., 2020). Moreover, AVP is present in the CSF of patients diagnosed with neurological diseases, such as organic dementia, Parkinson’s disease, and MS (Sundquist et al., 1983). Viñuela-Berni et al. (2020), using the EAE rat model, demonstrated the pivotal role of increased AVP serum levels on BBB permeability, whereas Quintanar-Stephano et al. (2012, 2019) showed the exacerbation of EAE clinical symptoms using desmopressin (dDAVP, a synthetic agonist of the V2 AVP receptors) in low AVP serum levels NIL rats. This is evaluated by the effects of desmopressin, which increases the permeability of the BBB membrane. Evidence that AVP deficiency is responsible for the decreased immune response in the EAE rats is strongly supported by the similar decreased severity in the clinical symptoms and brain and spinal cord histopathological lymphocyte infiltrations with conivaptan, a specific V1a-V2 AVP receptors antagonist (Quintanar-Stephano et al., 2012; Quintanar-Stephano et al., 2019, 2018; Viñuela-Berni et al., 2020; Figure 2). On the other hand, Bossinger suggested that the administration of OXT in patients with MS during aggravation or remission periods alleviates the symptoms (Bossinger, 1966); however, AVP and OXT can bind to V1a and V2 receptors (Terrillon et al., 2003). In 2014, a study by Verdugo-Meza et al. (2014) using NIL rats immunized for EAE compared the effects of OXT and desmopressin treatments. The results showed a significant increase in the CNS lymphocytic infiltration in rats treated with desmopressin, compared to OXT treated animals. Taken together, this evidence suggests that AVP deficiency or the blockade of V1a-V2 AVP receptors may be considered a new therapeutic strategy for MS.

### 1.3. AVP hormone

#### 1.3.1. Pleiotropic effects of AVP

Arginine vasopressin is synthesized primarily in the magnocellular neurons of the hypothalamic paraventricular and supraoptic nuclei as pre-pro-AVP, is cleaved into AVP, copeptin, and neurophysin II, which are axonally transported to the posterior pituitary and released into the general circulation (Davies et al., 2003; Quintanar-Stephano et al., 2012; Wasilewski et al., 2016).

Arginine vasopressin regulates various physiological functions, of which the most well-known are osmotic balance and vasopressor action in response to increased serum osmolality and decreased blood volume and pressure (Mavani et al., 2015).

#### 1.3.2. AVP receptors

Arginine vasopressin acts through three specific cell membrane receptors: V1a, V1b (also called V3), and V2, which are expressed in several tissues (Quintanar-Stephano et al., 2012), are G-protein-coupled receptors (GPCRs), and activate different cell signaling pathways (Figure 2; Koshimizu et al., 2012) as follows:

##### 1.3.2.1. V1a receptor (V1aR)

Ligand binding to this GPCR triggers the Gq protein pathway, inducing downstream phospholipase C (PLC) activation, leading to inositol triphosphate (IP-3), diacylglycerol (DAG) formation, and protein kinase C (PKC) activation (Wasilewski et al., 2016). These receptors are expressed mostly on the myometrium and vascular smooth muscle cells, where they induce myo-and vasoconstriction, respectively. V1aR activation mediates glycogenolysis and platelet aggregation by its expression on hepatocytes and platelets (Figure 2; Rotondo et al., 2016), as well as the regulation of bone formation and absorption *via* osteoblasts and osteoclasts, respectively (Tamma et al., 2013). Antagonists of V1aRs suppress cell cycle progression in the mesangial, vascular smooth, rat intestinal epithelial, cardiac, and liver parenchymal cells (Ghosh et al., 2001; Thibonnier et al., 2001; Chiu et al., 2002; Koshimizu et al., 2012). In addition, small cell lung cancer expresses V1aR and can secrete neuropeptides that are related to chemoresistance to the cancer drug etoposide (MacKinnon et al., 2009).

Other AVP effects include a decrease in pain when administered intracerebroventricularly (ICV). V1aRs are also expressed in the neuronal cells through the hypothalamus, amygdala, lateral septum, and other structures (Bodnar et al., 1982; Mavani et al., 2015). Hiroyama et al. (2007) demonstrated the role of V1aR in lipolysis regulation as an antilipolytic agent. V1aR is also associated with autism and induces impaired social behavior, anxiety, and memory. V1aR null mice and microinjections of a V1aR antagonist in rats reduced memory and anxiety, respectively. These results strongly suggest a role for AVP in social behavior (Heinrichs et al., 2009).

##### 1.3.2.2. V1b receptors (V1bR)

The stimulation of this receptor activates PLC, inducing increases in cytosolic calcium and the activation of PKC (Carvallo and Aguilera, 1989; Volpi et al., 2006). These receptors are mainly expressed in the anterior pituitary, adrenal medulla, white adipose tissue, islet cells of Langerhans, lateral septum, hippocampus, and amygdala, among other structures, and have been related to stress and pain perception. The AVP effect on corticotroph cells of the anterior lobe of the pituitary gland in response to psychosocial stress, and cognitive functions increases cortisol levels and is considered a key player in the development of metabolic syndrome. OXT is an anxiolytic hormone, whereas AVP is anxiogenic, and an imbalance between these hormones has been linked to various mental disorders such as anxiety, depression, schizophrenia, and autism (Mavani et al., 2015).

##### 1.3.2.3. V2 Receptor (V2R)

This receptor is a GPCR Gs. AVP binding generates cyclic adenosine monophosphate (cAMP) *via* the adenylate cyclase, activation of protein kinase A (PKA), and calcium mobilization (Yip, 2002). V2Rs are expressed in the kidney loop of Henle and the basolateral membrane of the collecting tubules, and it is responsible for the insertion of aquaporin-2 (water channels), favoring water reabsorption (antidiuretic effect). In vascular endothelial cells, AVP interacts with V2Rs, stimulating the release of the von Willebrand factor and factor VIII, affecting platelet aggregation and factor VIII in the coagulation cascade (Rotondo et al., 2016; Figure 2).

#### 1.3.3. AVP in immune responses

The expression of AVP receptors in immune cells has been demonstrated, as well as its role in immunological processes, although the molecular mechanisms of action have been poorly explored. Some studies have demonstrated the expression of AVP receptors in immune cells using radiolabeled ligands to confirm their presence in human mononuclear phagocytes, whereas its hormone activity was evaluated using vasopressin analogs. In 1983, Locher et al. (1983) described in *in vitro* assays the increase in prostaglandin E2 (PGE2) levels in human mononuclear phagocytes in response to AVP and desmopressin (dDAVP) suggested the presence of V2 AVP receptors (Quintanar-Stephano et al., 2012). The effect of PGE2 on cAMP metabolism increased 5- to 7-fold in the presence of AVP, and the presence of AVP receptors in PBMCs is associated with increased lymphocyte proliferation in the presence of increased AVP concentrations, demonstrating the immunoregulatory role of AVP (Locher et al., 1983; Quintanar-Stephano et al., 2016). On the other hand, AVP deficiency can induce disorders associated with immune response; for instance, the Brattleboro rat strain developed diabetes insipidus (DI) because of mutations in genes that regulate the production of AVP. These animals showed a significant decrease in the number of blood lymphocytes, macrophage activity, and dysregulation of principal lymphoid organs [revised in Quintanar-Stephano et al. (2016)].

The role of AVP as an anti-inflammatory hormone has been suggested by several authors. Zhao (2004) demonstrated that the activation of V1aR promotes the suppression of pro-inflammatory cytokines such as IL-1β and TNF-α in the astrocytes dependent on CREB (transcription factor associated with response of cyclic AMP) activation, suggesting that V1aR indirectly promotes neuroprotection of the cortical neurons (Zhao, 2004), and that the immunoregulatory effect of AVP hormone plus corticotrophin-releasing hormone (CRH) has been associated with the downregulation of splenic T cells and NK cells mediated by response to stress stimuli; however, that condition is reversible with the use of V1aR antagonist (Shibasaki et al., 1998; Russell and Walley, 2010). The V1aR can be expressed in PBMCs, mainly in monocytes and immune tissues (thymus and spleen), and its expression can be influenced by reproductive hormones. Thus, the expression of AVP receptors can be seven times higher in females than in males, and these differences have been associated with the role of estrogens in the production and release of AVP and regulation of their receptors (Elands, 1990; Sladek and Somponpun, 2008). In several diseases, E2 is a potential activator or inhibitor of pathophysiological disorders. Three-quarters of patients with MS are women (Reich et al., 2018; Wallin et al., 2019) and E2 acts as a protective factor by promoting the expression of anti-inflammatory cytokines such as IL-4 and IL-10 and the activation of Treg cells, with the promotion of Th2 phenotypic cells, protecting oligodendrocytes from cytotoxicity. However, during the sexual cycle, when E2 concentrations are lower, pro-inflammatory cytokines such as TNF-α, INF-γ, and IL-12 stimulate NK cell activity (Tomassini, 2005; Deckx et al., 2013; Villa et al., 2015). This may be the main reason why MS in both women and men develops at similar rates of progression and show poorer prognosis at >50 years (Miller et al., 2014; Ysrraelit and Correale, 2019).

In addition, hormonal stress response is regulated by the hypothalamic-pituitary-adrenal (HPA) axis and collaborates with the IS to regulate several physiological functions through negative feedback between the components of the neuro-immuno-endocrine axis. In MS, the secretion of AVP and CRH is closely related to the promotion of the disease and the co-expression of CRH/AVP in the hypothalamic neurons is higher in patients with MS than in the controls (Deckx et al., 2013).

Furthermore, some PBMCs express both AVP and CRH (Reder, 1992; Russell and Walley, 2010) and are also expressed in T cells, B cells, and macrophages, suggesting a possible relationship between chronic stress and autoimmune diseases, as it has been described in central diabetes insipidus (lack of AVP) and RA, where the presence of AVP is considered an inductor of inflammatory disorders (Elands, 1990; Patchev et al., 1993; Bellis et al., 1994; Chikanza and Grossman, 1998; Pivonello et al., 2003; Russell and Walley, 2010). Moreover, AVP participates in the enhancement of inflammatory responses of the Lewis strain rat, as described in 1993 by Patchev et al. (1993) who demonstrated high amounts of AVP (mRNA concentrations in the supraoptic and paraventricular nuclei), whereas CRH decreased in the paraventricular nuclei, increasing the susceptibility to develop inflammatory foci in Lewis strains. On the other hand, the F344/N strain showed opposite differences in the mRNA levels of both neuropeptides, suggesting that the F344/N strain could be more resistant to the inflammation process. This evidence supports the hypothesis that AVP acts as a potential proinflammatory agent in several inflammatory diseases (Patchev et al., 1993).

#### 1.3.4. Role of AVP hormone in the development of EAE

The neuroendocrine system effect on immune and inflammatory responses via the pituitary hormones, growth hormone (GH) and prolactin (PRL), as immunomodulators, and the hypothalamic-pituitary-adrenal (HPA) axis, are well-known. AVP is a pleotropic hormone that participates in water regulation, vasoconstriction, bone formation, glycogenolysis, platelet aggregation, antilipolytic effects, and impaired social behavior; however, the effects of AVP on the IS remains unclear. Evidence indicates that AVP plays a key immunostimulating role as an immune regulator in *in vitro* (autologous lymphocytes and splenocytes) and *in vivo* models (Quintanar-Stephano et al., 2016).

**FIGURE 3:**
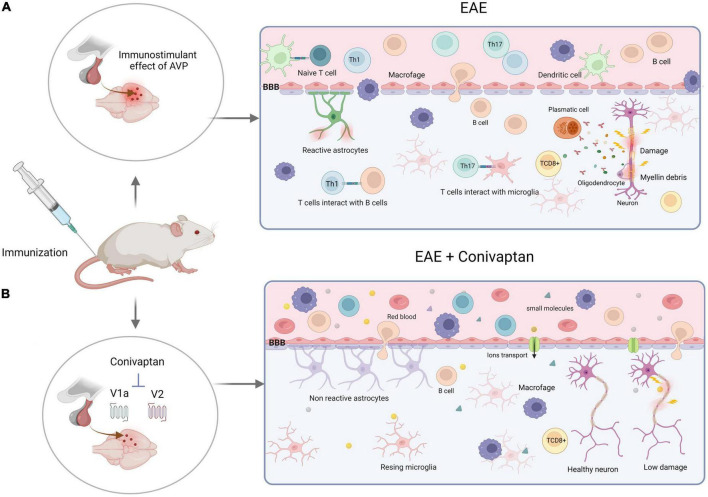
Schematic illustration of experimental autoimmune encephalomyelitis (EAE) development with V1a-V2 arginine vasopressin (AVP) receptor blocker. **(A)** Encephalitogenic immunization of the rats trigger immune response through the activation of autoreactive T cells by dendritic cells. The clonal expansion takes place in the lymph nodes inducing cell differentiation (Th1 and Th17). At the same time, the neurohypophysial secretion of AVP affects vascular permeability of the blood-brain barrier (BBB) through the action on its receptors (V1aR and V2R), increasing the production of proinflammatory cytokines, autoantibodies and CD8+ T cells that induce axonal and myelin damage and destruction. **(B)** Blockade of the V1a and V2 receptors of AVP by conivaptan diminish the autoimmune, proinflammatory responses and BBB permeability. Thus, protecting the animals against developing severe EAE disease. Created with BioRender.com.

Experimental autoimmune encephalomyelitis is an MS experimental model that causes brain and spinal cord inflammation mediated by Th1 and Th17 cells and induces axonal myelin loss mediated by activated T lymphocytes against oligodendrocytes. In more advanced stages, the animals develop hind leg paralysis. Activation of AVP receptors expressed in immune cells increases the levels of pro-inflammatory cytokines (IL-2, IL-6, IL-17, INF-γ, and TNF-α), whereas AVP deficiency (NIL-induced) or blocking of the AVP receptors with a V1a and V2 AVP antagonist (conivaptan) decreases clinical symptoms, pro-inflammatory cytokines, and BBB permeability (Figure 3; (Quintanar-Stephano et al., 2018; Viñuela-Berni et al., 2020).

#### 1.3.5. AVP receptors in the nervous system

##### 1.3.5.1. V1aRs

Studies have indicated the broad expression of V1aR in the brain and spinal cord cells. In humans and rodents, they are extensively located in the cerebral cortex, hippocampus and choroid plexus (Ostrowski et al., 1994; Campbell et al., 2009). The mRNA V1aRs are also highly expressed in the astrocytes, perivascular smooth muscle, and endothelial vascular cells, suggesting a regulatory role for AVP in vascular resistance, cerebral circulation, and brain water homeostasis (Szmydynger-Chodobska et al., 2004; Kozniewska and Romaniuk, 2008).

In MS, the BBB is permeable to immune cell infiltration, mainly CD4+ and CD8+ T cells, in the brain and spinal cord perivascular spaces (Babbe et al., 2000; Alvarez et al., 2011; Lassmann and van Horssen, 2011). In EAE, BBB disruption is related to diminished expression of claudin-5, which facilitates the inflammatory process in the spinal cord and brain, as demonstrated by Viñuela-Berni et al. (2020) who described the role of V1aR and V2R in the permeability of the BBB and immune cell infiltration in the EAE model, an event related to the inflammatory pathogenic process in MS, which was significantly decreased in the EAE rats subjected to NIL or conivaptan treatment (the V1a-V2 AVP receptor antagonist) (Viñuela-Berni et al., 2020).

##### 1.3.5.2. V1bRs

These receptors are mainly expressed in the anterior pituitary, adrenal medulla, white adipose tissue, pancreatic islets of Langerhans, lateral septum, hippocampus, and amygdala, among other structures. These receptors are involved in stress and pain perception. AVP plays an important role in the HPA system during stress conditions. Psychosocial stress induces an increase in serum cortisol levels, which play a key role in the development of metabolic syndrome (Mavani et al., 2015). V1bR is expressed in immune cells (Lolait et al., 1995); however, its role in immunoregulation in EAE has not been studied.

##### 1.3.5.3. V2Rs

Yamaguchi et al. (2004) demonstrated that V2R expression in lymphocytes and its activation by [deamino-Cys(l), d-Arg(8)]-vasopressin (dDAVP) induces fibrinolytic activity in these cells (Yamaguchi et al., 2004). Desmopressin is a synthetic analog of AVP for V2R. It restores or enhances the levels of IL-1 and IL c-2 in splenic ells in NIL animals, increasing the stimulation of immune response in EAE animals, and conivaptan (a V1aR and V2R antagonist) diminishes the activity of the IS in EAE (Quintanar-Stephano et al., 2018).

#### 1.3.6. Ligands of AVP receptors

Some ligands interact specifically with AVP receptors, acting as specific agonists and antagonists. This specificity and affinity are therapeutic strategies for treating some pathological diseases.

##### 1.3.6.1. Agonist of AVP

Arginine vasopressin binding to its receptors induces the physiological effects as mentioned earlier in “section 1.3.1 Pleiotropic effects of AVP.” However, its peptide nature allows it to be a target of rapid metabolism and excretion (half-life in plasma is <24 min) (Baumann and Dingman, 1976), making the use of AVP as a tool for peripheral or potential therapeutic effects challenging (Manning et al., 2012). The generation of peptides and non-peptides AVP receptor ligands has become a critical tool for achieving these objectives.

##### 1.3.6.2. Terlipresin

A synthetic AVP analog with vasoconstrictor activity in both the vascular and splanchnic systems (Jamil et al., 2017) has proven that the interaction of terlipressin and V1aR induces the reduction of portal vein hypertension by diminishing portal blood flow. Owing to these effects, terlipressin plus albumin has been proven to be an effective drug for the treatment of type 1 hepatorenal syndrome in patients with cirrhosis (Wong et al., 2021). Several studies have demonstrated the possible beneficial effects of terlipressin as an alternative to norepinephrine (NE)-resistant refractory septic shock; however, the continuous infusion of terlipressin is limited because of adverse effects such as digital ischemia, arrhythmia, acute mesenteric ischemia, and hyponatremia, with no significant difference in the 28 days mortality rate between NE and terlipressin (Study Group of investigators et al., 2018). Terlipressin-induced amelioration of myocardial contractility, renal function, and vascular leakage in septic shock has been suggested in animal models (Morelli et al., 2004; Rehberg et al., 2009; Lange et al., 2011).

##### 1.3.6.3. Felypressin

Since the 1970s, this synthetic analog of AVP, with high affinity to V1aR, has been used as an adjuvant in nerve blockage in dental procedures because of its vasopressor effect, prolonging the anesthetic effect of anesthesia (Shinzaki and Sunada, 2015). However, recent studies suggested a possible contraindication of this drug in hypertensive patients owing to an increase in diastolic pressure in patients with controlled blood pressure (Bronzo et al., 2012).

##### 1.3.6.4. Desmopressin (dDAVP)

Also known as 1-deamino-8-D-arginine vasopressin, is a synthetic analog of vasopressin for treating hemostatic diseases such as hemophilia A, von Willebrand disease, and uremic bleeding. In addition, it is used off-label in trauma resuscitation with active and intracranial hemorrhage associated with varying antiplatelet agents through its role as releaser of von Willebrand factor *via* V2R agonism (Ozgonenel et al., 2007). Because of its antidiuretic effect, it is used in diabetes insipidus, nocturnal polyuria, and other conditions, such as supplementation with hypertonic saline to prevent rapid sodium correction (McCarty and Shah, 2022). Studies on patients with melancholic depression have demonstrated increased adenocorticotropic hormone (ACTH) release after desmopressin administration through hyper-responsiveness to the activation of V1bR, showing a mixed agonism of this compound, which was blocked by selective V1bR antagonist administration (Craighead et al., 2008). The immunostimulating effect of desmopressin has been demonstrated in NIL rats immunized for EAE, in this animal model of MS, desmpressin treatment causes worse development in clinical and hystophatological signs of EAE (Quintanar-Stephano et al., 2012; Viñuela-Berni et al., 2020).

##### 1.3.6.5. Antagonists of AVP

Arginine vasopressin has pleiotropic effects owing to its affinity to the three known AVP receptors and the various tissues where they are expressed. In addition to the increase in AVP plasma levels in many pathologies such as anxiety, congestive heart failure, hyponatremia, edematous diseases, immune responses, among others (Ishikawa, 2017), the importance of specific receptors blockers and their key role in the modulation of diverse pathologies have been highlighted below.

##### 1.3.6.6. SRX246

It is a novel V1aR antagonist that shows a good anxiolytic effect, although it is not yet approved to be used in humans. In pre-clinical studies, this compound has been permitted to validate the AVP role in the regulation of fear and anxiety in humans (Lago et al., 2021).

##### 1.3.6.7. Vaptans

This is a family of AVP antagonist with different receptor affinities, whose use has been limited to cardiac conditions, osmolarity, and advanced liver cirrhosis.

The vaptans approved for human use are discussed below.

###### 1.3.6.7.1. Tolvaptan

It is a selective V2R blocker. Its primary pharmacological effect is kidney aquaresis (loss of water without ions in urine) without modification of renal hemodynamics, which means the maintenance of renal blood flow, neurohormones, and plasma electrolyte concentrations (Costello-Boerrigter et al., 2006). The Food and Drug Administration (FDA) approved tolvaptan as Samsca™ for heart failure; however, there was a warning that Samsca should not be administered for more than 30 days because of possible liver injury (Food and Drug Administration, 2013).

###### 1.3.6.7.2. Conivaptan

This vaptan competitively and reversibly inhibits vasopressin binding to V1aR and V2R. Its effects are dose-dependent, increased urine excretion, it reduces the pulmonary capillary wedge pressure (PCWP) and sustaining right atrial pressure for 12 h without changes in cardiac index, systemic or pulmonary vascular resistance, blood pressure, or heart rate (Udelson et al., 2001). In addition, its aquaretic effect prevents electrolyte loss in urine compared to conventional diuretics (Urbach and Goldsmith, 2021). Currently, conivaptan is approved by the FDA as Vaprisol™ for the treatment of euvolemic hyponatremia in patients under medical supervision. Presently, positive results in heart failure with conivaptan have been demonstrated; however, the FDA has not yet approved its use (ASTELLAS Pharma US, Inc., 2005).

###### 1.3.6.7.3. Mozavaptan

It is known as OPC-31260. It has been shown to have pharmacological effects experimentally by inducing endolymphatic hydrops in guinea pigs, a Meniere’s disease model. It is a promising drug because of the significant decrease in the ratio of aquaporin 2 (AQP2) to α-actin mRNA in the cochlea and endolymphatic sac (Takeda et al., 2003). This compound is the first non-peptidyl oral V2R blocker and was approved for the treatment of hyponatremia in paraneoplastic syndrome of inappropriate antidiuretic hormone secretion (SIADH) by the Pharmaceuticals and Medical Devices Agency (Otsuka Pharmaceutical Co., Ltd., 2006).

###### 1.3.6.7.4. Recolvaptan

A V1aR blocker that is in the clinical phase of development. It has a dose-dependent inhibitory effect on uterine contractions (Sanofi-Synthélabo, n.d.; Zhao et al., 2018) and its effect on V1aR blockage in castration-resistant prostate cancer (CRPC), showed an inhibitory effect on the proliferation and metastatic features of CRPC cells in both *in vivo* and *in vitro* studies (Zhao et al., 2018).

In summary, vaptans are well-tolerated drugs; however, they have secondary effects such as thirst, pollakiuria, dry mouth, hypernatremia, or rebound hyponatremia after withdrawal treatment, and their use is restricted to euvolemic hyponatremia caused by emetic stimuli and secondary adrenal insufficiency, among others (Aditya and Rattan, 2012).

## 2. Conclusion

Multiple sclerosis is a multifactorial disease associated with environmental, genetic, epigenetic, and immunological processes. Currently, the use of conventional treatments continues to be a subject of study to determine the most appropriate therapy for each patient. However, in most cases, the use of specific and targeted treatments is not available to all patients, and the adverse effects associated with their use makes it difficult to improve symptoms and relapse episodes. In this review, we proposed the use of V1a and V2 blockers of AVP such as conivaptan which acts as an immune modulator. Its use can reduce the exacerbated response of the immune system without completely depleting its activity; therefore, this treatment can provide a better quality of life, with less secondary effects, as observed in studies carried out in animal models of EAE. It is expected that in the future, research on the effects of agonists and antagonists of the different AVP receptors will result in major advances in the understanding and treatment of many diseases associated with the immune system. This new perspective opens a new panorama for the treatment of patients diagnosed with MS.

## Author contributions

AC-R, AQ-S, and CR-F designed the original idea and developed the central content of the research. AC-R and CR-F designed the tables and figures. FV-U and EH-C participated in the manuscript preparation and correction. All authors read and approved the final version.
